# Solid Solutions of Lindbergite–Glushinskite Series: Synthesis, Ionic Substitutions, Phase Transformation and Crystal Morphology

**DOI:** 10.3390/ijms232314734

**Published:** 2022-11-25

**Authors:** Anatolii V. Korneev, Alina R. Izatulina, Mariya A. Kuz’mina, Olga V. Frank-Kamenetskaya

**Affiliations:** Crystallography Department, Institute of Earth Sciences, Saint-Petersburg University, 7/9 Universitetskaya nab., 199034 St. Petersburg, Russia

**Keywords:** lindbergite, glushinskite, humboldtine, X-ray diffraction, solid solutions, ionic substitutions, X-ray powder diffraction, scanning electron microscopy, EDX spectroscopy

## Abstract

To clarify the crystal chemical features of natural and synthetic oxalates *Me*^2+^(C_2_O_4_)∙2H_2_O (*Me*^2+^ = Fe, Mn, Mg, Zn), including minerals of the humboldtine group, solid solutions of lindbergite Mn(C_2_O_4_)∙2H_2_O–glushinskite Mg(C_2_O_4_)∙2H_2_O were precipitated under various conditions, close to those characteristic of mineralization in biofilms: at the stoichiometric ratios ((Mn + Mg)/C_2_O_4_ = 1) and non-stochiometric ratios ((Mn + Mg)/C_2_O_4_ < 1), in the presence and absence of citrate ions. Investigation of precipitates was carried out by powder X-ray diffraction, scanning electron microscopy and energy-dispersive X-ray spectroscopy. Thermodynamic modelling was performed in order to evaluate the lindbergite–glushinskite equilibrium. It was shown that glushinskite belongs to the orthorhombic β-modification (sp. Gr. *Fddd*), while lindbergite has a monoclinic α-modification (sp. gr. *C*2/*c*). Mg ions incorporate lindbergite in much higher quantities than Mn ions incorporate glushinskite; moreover, Mn glushinskites are characterized by violations of long-range order in their crystal structure. Lindbergite–glushinskite transition occurs abruptly and can be classified as a first-order isodimorphic transition. The Me^2+^/C_2_O_4_ ratio and the presence of citric acid in the solution affect the isomorphic capacity of lindbergite and glushinskite, the width of the transition and the equilibrium Mg/Mn ratio. The transition is accompanied by continuous morphological changes in crystals and crystal intergrowths. Given the obtained results, it is necessary to take into account in biotechnologies aimed at the bioremediation/bioleaching of metals from media containing mixtures of cations (Mg, Mn, Fe, Zn).

## 1. Introduction

Manganese and magnesium oxalate dihydrates (minerals lindbergite Mn(C_2_O_4_)∙2H_2_O and glushinskite Mg(C_2_O_4_)∙2H_2_O) belong to the humboldtine group, along with iron and zinc oxalates (minerals humboldtine and katsarosite) [1]. They were first found in lichens: Mg oxalate in cretaceous coals (1960 or earlier) [2] and later on serpentinites [3]; Mn oxalate on manganese ore containing manganese oxides (lithiophorite, cryptomelane, hollandite) in 1984 [4]. These oxalates were additionally investigated and approved as new minerals in 1980 [3] and 2004 [5], respectively. For a long time, all minerals of the humboldtine group were considered monoclinic (sp. gr. *C*2/*c*) and isotypic [5].

As was shown by the investigation of synthetic compositions, oxalates *Me*^2+^(C_2_O_4_)·2H_2_O (*Me*^2+^ = Fe, Mn, Mg and other) can exist in several polymorphic modifications ([6], Table 1). Moreover, it is known that transition metal oxalate dihydrates have a tendency to form disordered structures [7].

According to the earliest powder X-ray diffraction (PXRD) data [8], Mg oxalate was proposed to exist in *α*- and *β*-modifications (ordered and disordered, respectively), which are characterized by the same monoclinic symmetry (sp. gr. *C*2/*c*). The existence of a disordered modification of Mg oxalate dihydrate was confirmed later by single crystal X-ray photographs [9]. Wilson and co-authors have reported mineral glushinskite as a natural analogue of disordered monoclinic *β*-modification [3]. Later, Chen and co-authors performed a single-crystal X-ray diffraction study revealing the orthorhombic symmetry of *β-*MgC_2_O_4_∙2H_2_O with space group *Fddd*, of which *C*2/*c* is a subgroup [10]. Defectiveness was not discussed in this study.

**Table 1:** Symmetry and unit cell parameters of natural and synthetic oxalates Me^2+^(C_2_O_4_)∙2H_2_O, *Me* = Mn^2+^, Mg^2+^.

Compound	Sp.gr. sym.	a, Å	b, Å	c, Å	β, °	Reference
Lindbergite	*C*2/*c*	11.995(5)	5.632(2)	9.967(7)	128.34(4)	[5]
*α*-MnC_2_O_4_·2H_2_O	*C*2/*c*	12.016	5.632	9.961	128.37	[11], PDF #00-025-0544 ***
*α*-MnC_2_O_4_·2H_2_O	*C*2/*c*	11.765(2)	5.655(1)	9.637(1)	125.84(1)	[12]
*α′*-MnC_2_O_4_·2H_2_O	*C*2/*c*	11.998(4)	5.647(6)	9.985(3)	128.34(4)	[7]
*α″*-MnC_2_O_4_·2H_2_O	*C*2/*c*	11.939(5)	5.624(7)	9.703(3)	126.52(6)	[7]
*γ*-MnC_2_O_4_·2H_2_O	*P*2_1_2_1_2_1_	6.262(4)	13.585(5)	6.091(4)	90	[13]
Glushinskite *	*C*2/*c*	12.688	5.400	9.959	129.44	[3]
*α*-MgC_2_O_4_·2H_2_O	*C*2/*c*	12.689	5.391	9.977	129.82	[8], PDF #00-026-1223 ***
*β*-MgC_2_O_4_·2H_2_O	*C*2/*c*	12.675	5.406	9.984	129.45	[8] ***
*β*-MgC_2_O_4_·2H_2_O	*Fddd* **	12.691(3)	5.394(1)	15.399(3)	90	[10]

* Calculated with UnitCell [14] based on author’s indexes and d-spacings. ** Crystal setting was changed for comparison with monoclinic species. *** Errors of unit cell parameters are not given as they were not present originally.

Mn oxalate dihydrate can crystallize in monoclinic α-modification (sp. gr. *C*2/*c*) [11,12] and in orthorhombic γ-modification (sp. gr. *P*2_1_2_1_2_1_) [13], as follows from single-crystal X-ray diffraction data. Deyrieux with co-authors and Puzan with co-authors have also shown that α-MnC_2_O_4_·2H_2_O can exist in two variants (α′ and α″) having the same symmetry (sp. gr. *C*2/*c*) and different unit cell parameters [7,11]. Based on XRPD data, lindbergite is a natural analogue of *α*-modification [5].

**Figure 1:**
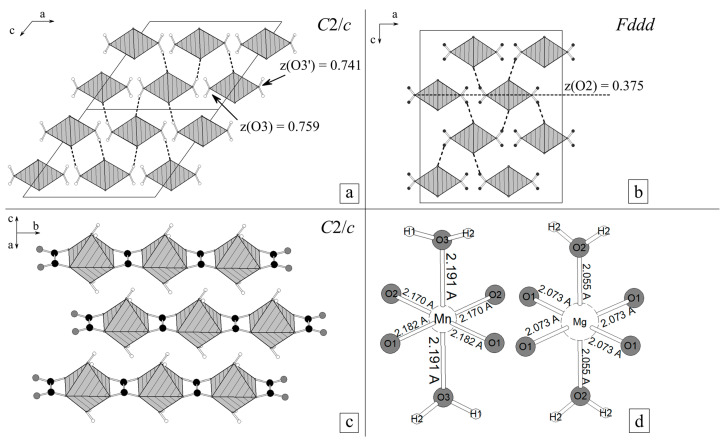
Crystal structures of Mn and Mg oxalate dihydrates: (**a**)—chains of *Me*^2+^ octahedra and oxalate ions along [010] (by example of *α-*MnC_2_O_4_·2H_2_O, [12]); (**b**)—octahedra *Me*^2+^O_4_(H_2_O)_2_; (**c**)—projection of structure of *α*-MnC_2_O_4_·2H_2_O on ac plane; (**d**)—projection of structure of *β*-MgC_2_O_4_·2H_2_O on ac plane ([10], a↔b). Notation: hatched octahedra—*Me*^2+^O_4_(H_2_O)_2_; ●—carbon atoms; ○—hydrogen atoms; black dashed lines—hydrogen bonds.

Crystal structures of monoclinic α- and orthorhombic *β*-modifications are very similar according to a complex of structure data [10,12]. They contain chains of distorted octahedra *Me*^2+^O_4_(H_2_O)_2_ connected by flat oxalate ions [C_2_O_4_], acting as tetradentate ligands. The chains are parallel to the *b* axis (Figure 1a). Disordering manifestations are caused by the arbitrary displacements of the metal oxalate chains one to another [7,9,15]. The chains are connected by hydrogen bonds O-H···O, almost parallel to the ac plane. The similarity of the crystal structures of *α*- and *β*-modifications should contribute to unstudied yet ionic substitutions in minerals of the humboldtine group [16].

The crystal structure of *γ*-MnC_2_O_4_·2H_2_O [13] is significantly different from others by the construction of chains. In this case, octahedra are linked by shared oxygen atoms, as well as by oxalate ions, acting as tridentate ligands.

The formation of oxalates in the *Me*^2+^C_2_O_4_·2H_2_O (*Me*^2+^—Fe, Mn, Mg) system is important for study, primarily due to the interest in biotechnologies using a variety of microbes that leach manganese and other metals from ores, including poor ones. Biomining is an excellent green alternative to modern methods [17,18,19]. In bioleaching, microscopic fungi (micromycetes) are often used, which are active producers of various organic acids; then, bioleaching processes often occur through the formation of metal oxalates, such as manganese oxalates. For example, metal leaching from nodules in the Indian Ocean was performed using *Aspergillus niger* [17], manganese leaching from manganese ore using *Penicillium citrinum* and *Aspergillus* sp. [20] and the leaching of heavy metals from tailings with *Aspergillus fumigatus* [21].

Our study is devoted to patterns of structural relations and ionic substitutions in lindbergite–glushinskite series. In particular, our goals were to synthesize (Mg, Mn)C_2_O_4_·2H_2_O solid solutions in conditions close to those characteristic for biofilm mineral formation; to refine their structural modifications (as well as modifications of their natural analogues—minerals lindbergite and glushinskite); and to reveal the regularities of Mg incorporation in lindbergite/Mn incorporation in glushinskite and the influence of these substitutions on α-β transition and structural disorder, as well as the morphology of solid solution crystals.

## 2. Results

### 2.1. Powder X-ray Diffraction

#### 2.1.1. Evolution of Phase Composition of Precipitates on Change in Composition of the Initial Solution

**Figure 2:**
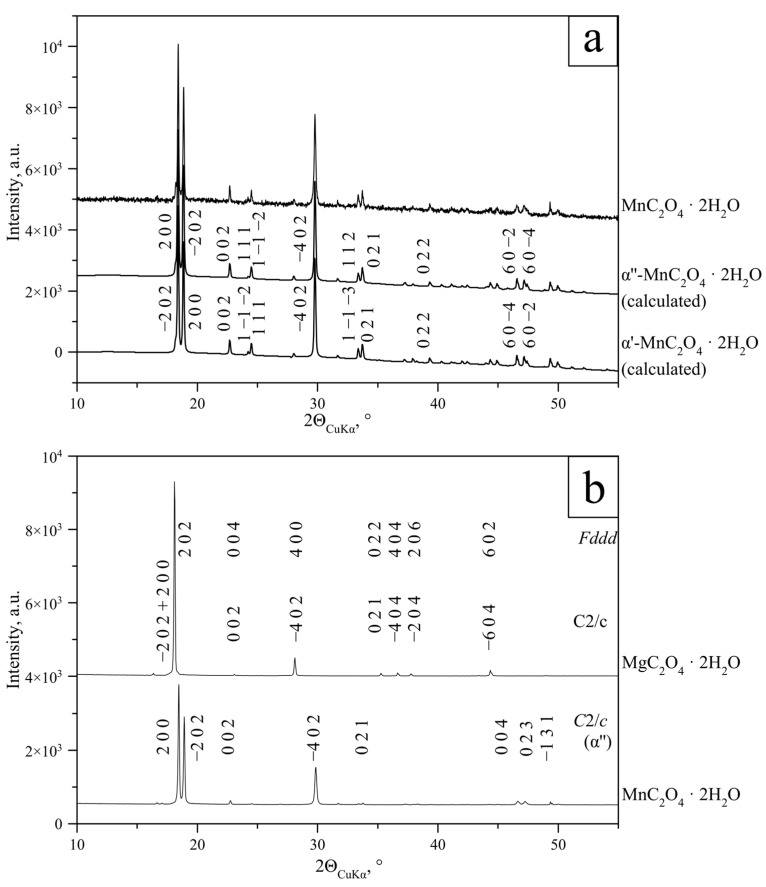
Results of indexing of end-members: (**a**)—MnC_2_O_4_·2H_2_O for *α′*- and *α″*-modifications (sp. gr. *C*2/*c*); (**b**)—MgC_2_O_4_·2H_2_O for *α*- and *β*-modifications (sp.gr. *C*2/*c* and *Fddd*, respectively). MnC_2_O_4_·2H_2_O pattern indexed in *C*2/*c* space group (α″-modification) is given for comparison.

Crystalline solid solutions (Mn, Mg) C_2_O_4_·2H_2_O were obtained in all syntheses with 0 < Mg/(Mg + Mn) < 1. In the case of MnC_2_O_4_·2H_2_O, indexing was successful in two variants corresponding to *α′*- and *α″*-modifications, and their calculated powder patterns matched completely (Table S1, Figure 2a). Thus, *α*’ and *α″* modifications of MnC_2_O_4_·2H_2_O cannot be distinguished through PXRD. For the determination of the unit cell parameters of lindbergites, we used setting matching *α″*-modifications as a priority, following Soleimannejad and co-authors [12], with a lower *c* parameter and a *β* angle closer to 90° (*α″*), which matches the IUCr recommendations.

In the case of MgC_2_O_4_·2H_2_O, indexing was successful in both *Fddd* and C2/c space groups (Table S2, Figure 2b). As the *C*2/*c* space group is a subgroup of *Fddd*, MgC_2_O_4_·2H_2_O is highly likely characterized by orthorhombic symmetry, but for an easier comparison of Mn and Mg oxalates’ powder patterns and unit cell parameters, from now on, we use the monoclinic indexing of MgC_2_O_4_·2H_2_O.

Comparison of the interplanar spacings (*d/n*) of synthesized phases and corresponding biominerals found in biofilms confirmed that the synthesized oxalates were analogues of lindbergite [4,5] and glushinskite [3].

**Figure 3:**
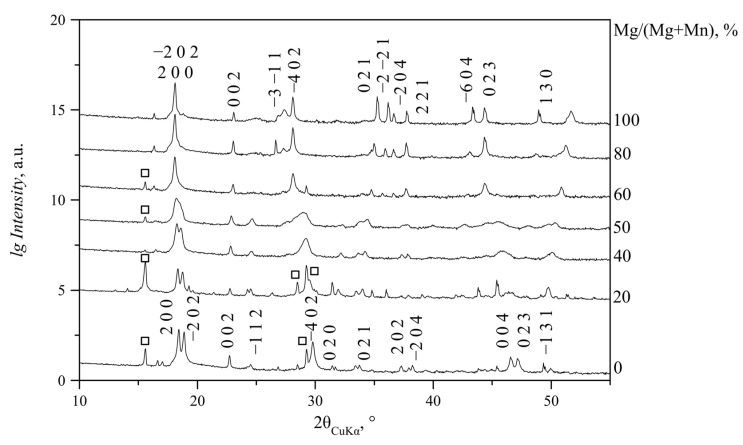
PXRD patterns of precipitates obtained at Mg/(Mg + Mn) = 0–100% in solution (NC series). □—peaks of MnC_2_O_4_·2H_2_O phase (Sp. gr. *P*2_1_2_1_2_1_). The remaining peaks belong to (Mn, Mg)C_2_O_4_·2H_2_O (Sp. gr. *C*2/*c*). As −2 0 2, 2 0 0 and −4 0 2 peaks’ intensities significantly exceed the intensities of the remaining peaks, intensities are given in logarithmic scale in Figure 3, Figure 4, Figure 5 and Figure 6.

**Figure 4:**
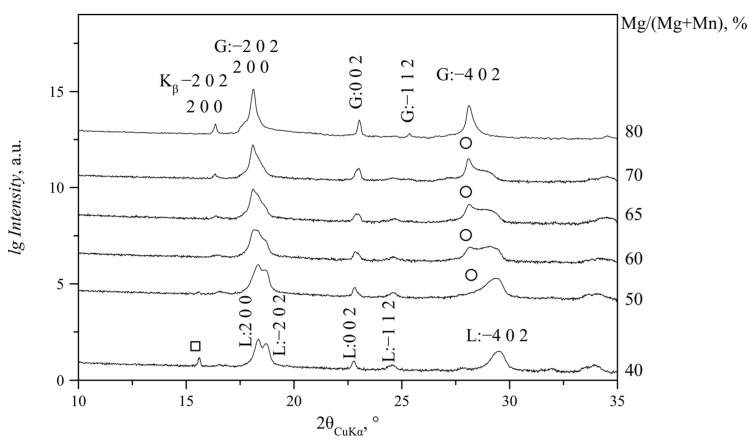
PXRD patterns of precipitates obtained at Mg/(Mg + Mn) = 40–80% (S series, 2θ_CuKα_ = 10–35°). Notation: L—solid solution with lindbergite structure; G—solid solution with glushinskite structure; □—peak of MnC_2_O_4_·2H_2_O phase (sp.gr. *P*2_1_2_1_2_1_); ○—peak indicating formation of solid solution with glushinskite structure.

For the Mg/(Mg + Mn) ratio in solution from 0 to 40% (N and NC series) or 50% (S and SC series), lindbergites were formed. With the increase in Mg content in solution, the slow broadening of the diffraction peaks takes place, and the peaks themselves shift in the direction of the glushinskite peaks (to a lower angle area predominantly) (Figure 3). Peaks 2 0 0 and −2 0 2 come closer at Mg/(Mg + Mn) = 40% at non-stochiometric *Me*^2+^/C_2_O_4_ ratio (N and NC series) and Mg/(Mg + Mn) = 50% at stochiometric ratio (S and SC series). At Mg/(Mg + Mn) = 50% (N and NC series) or 60% (S and SC series), these peaks merge into one asymmetric peak. The −4 0 2 peak becomes asymmetric at lower angles as well; with the increase in Mg content, an additional broad maximum (2θ_CuKα_ = 28.15°), characteristic of glushinskite, appears in its shoulder (Figure 4). With a further Mg content increase, this peak becomes sharper, while the lindbergite peaks continue to broaden. At Mg/(Mg + Mn) = 55–60%, the lindbergite −4 0 2 peak and newly formed peak (2θ_CuKα_ = 28.15°) are close in intensity. Further Mg content growth causes the broadening and fading of the lindbergite peaks, while the newly formed glushinskite peaks (2θ_CuKα_ = 28.15°, 36.60°, 37.70°, 44.34°) keep increasing and sharpening. The peak formed by the merging of 2 0 0 and −2 0 2 reflexes becomes symmetric. With Mg/(Mg + Mn) = 60–80%, the lindbergite peaks fade completely and only the glushinskite peaks remain. The lindbergite–glushinskite transition was the slowest in the S series (Mg/(Mg + Mn) = 45–75%) and the fastest in the NC series (Mg/(Mg + Mn) =45–55%).

**Figure 5:**
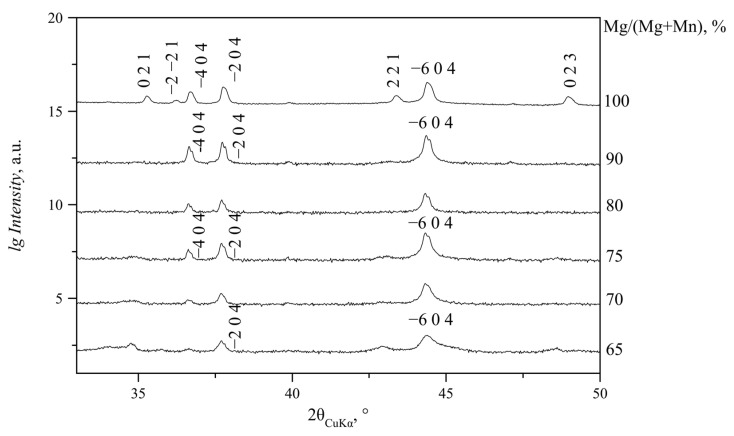
XRD patterns of precipitates obtained at Mg/(Mg + Mn) = 65–100% (N series, 2θ_CuKα_ = 33–50°).

XRD patterns of glushinskites at Mg/(Mg + Mn) in solution from 60 to 90% contain mainly *h0l* reflexes, which almost do not change their positions or intensities with the increase in Mg content. *hkl* reflexes are few; they are low in intensity and shift to high-angle areas with the increase in Mg content. On N series XRD patterns (Mg/(Mg + Mn) = 65–90% in solution), exceptionally, *h0l* reflexes are present (Figure 5).

**Figure 6:**
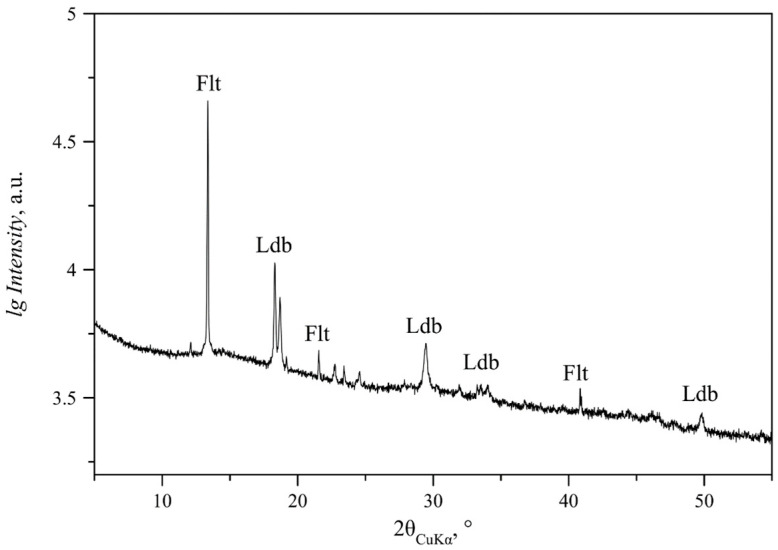
PXRD pattern of precipitate obtained at Mg/(Mg + Mn) = 30% (SC series). Notation: Flt—falottaite MnC_2_O_4·_3H_2_O; Ldb—solid solution with lindbergite structure.

Besides monoclinic Mg lindbergites and Mn glushinskites, trihydrate Mn oxalate (falottaite MnC_2_O_4_·3H_2_O, sp. gr. *Pcca*, ICSD #170689) and orthorhombic dihydrate Mn oxalate (MnC_2_O_4_·2H_2_O, sp. gr. *P*2_1_2_1_2_1_, ICSD#96426) were found in the precipitates. Falottaite was detected only in one sample of the SC series (Mg/(Mg + Mn) = 30%, Figure 6). Orthorhombic Mn oxalate was detected in the S and NC series, predominantly in the range of Mg/(Mg + Mn) from 0 to 40–60%.

#### 2.1.2. Variations in Unit Cell Parameters

**Figure 7:**
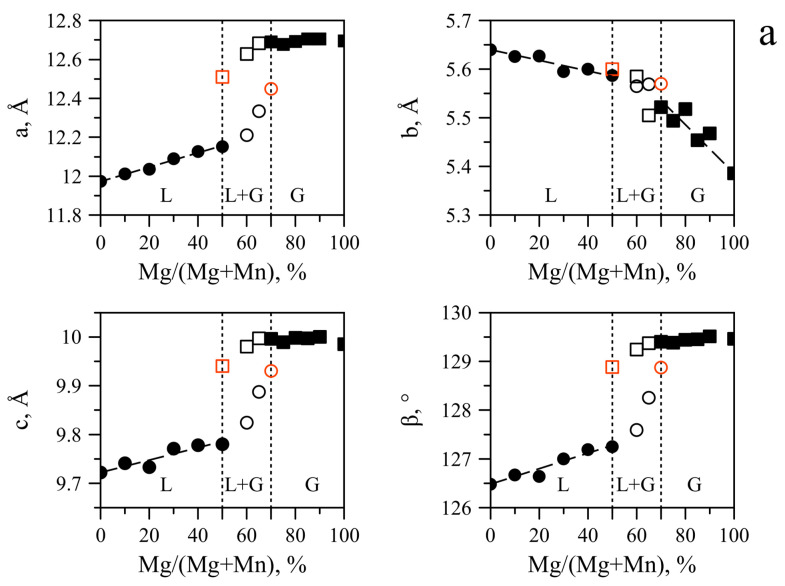

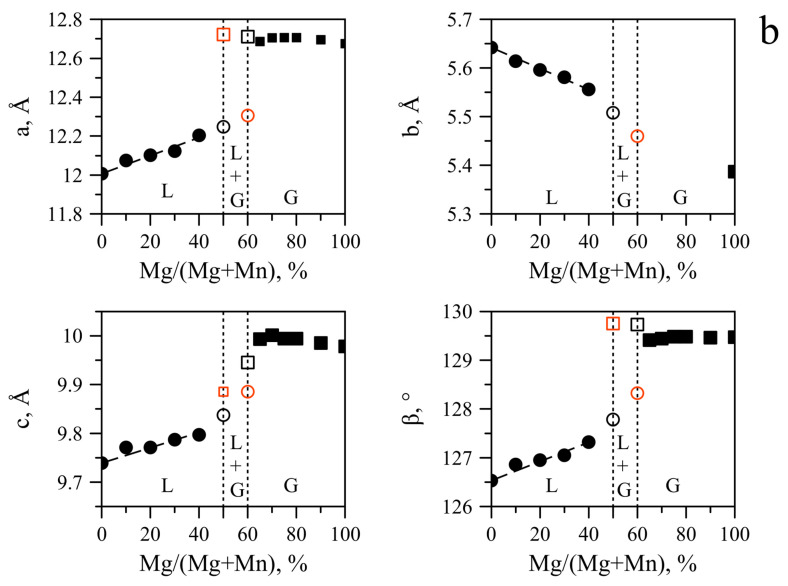

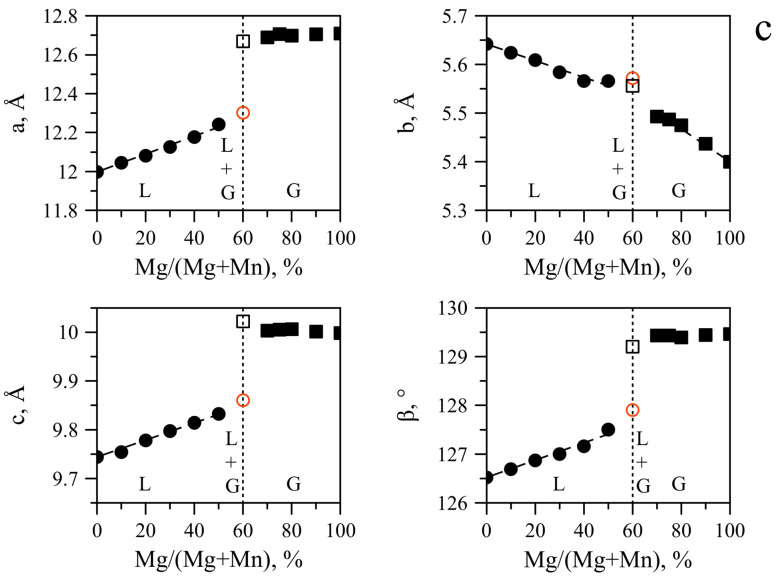

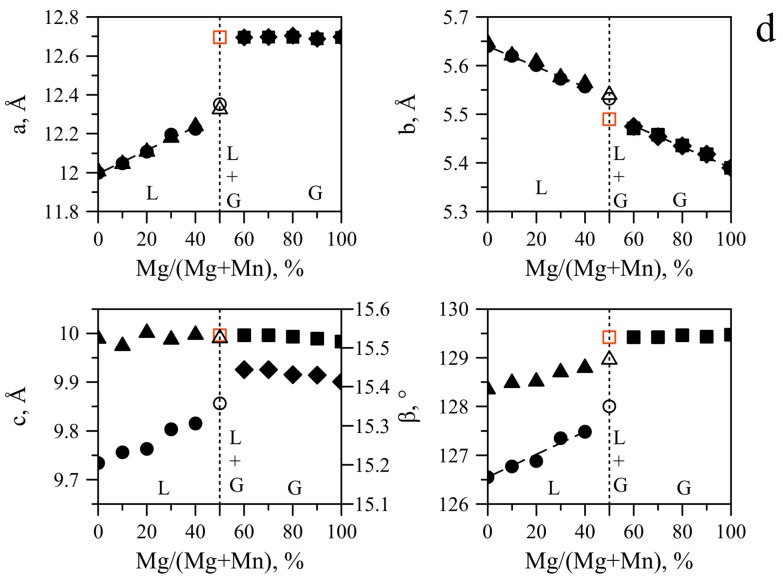
Unit cell parameters of lindbergite–glushinskite solid solution series depending on Mg/(Mg + Mn) ratio in solution obtained in different series: (**a**)—S; (**b**)—N; (**c**)—SC; (**d**)—NC. For NC series, comparison between monoclinic *α′* and *α″* settings (for lindbergite) and monoclinic and orthorhombic settings (for glushinskite) is given. For the remaining series, unit cell parameters are given for monoclinic setting (*α″* for lindbergite). Legend: ●○—lindbergites (monoclinic α″-modification); ▲△—lindbergites (monoclinic α′-modification); ■□—glushinskites (monoclinic setting); ⬥—glushinskites (orthorhombic β-modification). Errors of determination: bold markers—0.001 Å for linear parameters and 0.01° for *β*; black empty markers—0.01 Å for linear parameters and 0.1° for β; red markers—parameters were linearly extrapolated, error was not determined, the presence of corresponding phases is supported by X-ray phase analysis.

Unit cell parameters of lindbergites obtained from Mg-free solutions (α″ setting) vary as follows: *a* = 11.974 (S)—12.007 (N) Å; *b* = 5.640—5.642 Å; *c* = 9.722 (S)—9.744 (N) Å; *β* = 126.48 (S)—126.55 (NC)° (Figure 7). Variations in the *b* parameter are insignificant and do not exceed three standard errors (σ). Variations in other parameters are significant: *a*—up to 33σ, *c*—up to 22σ, *β*—up to 7σ. Parameters *a*, *c*, *β* of lindbergite obtained in the S series take the smallest values and significantly differ from those for other series. Parameters *a* and *c* of lindbergites reach maximum values in the N series and β in the NC series.

Unit cell parameters of lindbergites obtained from Mg-free solutions (α′ setting) vary as follows: *a* = 11.975 (S)—12.008 (N) Å; *b* = 5.641—5.643 Å; *c* = 9.973 (S)—9.989 (N) Å; *β* = 128.34 (NC)—128.51 (N)°. Parameters *a* and *b* stay the same as in the *α″* setting, and parameters *c* and *β* increase greatly.

The relation between parameters of two settings is *a_α′_* = *a_α″_*, *b_α′_* = *b_α″_*, *c_α′_*cos (*β_α′_*—90°) = *c_α″_*cos (*β_α″_*—90°).

Monoclinic unit cell parameters of glushinskites obtained under a lack of Mn vary as follows: *a* = 12.675 (N)—12.709 (SC) A; *b* = 5.386 (S, N)—5.400 (SC) A; *c* = 9.978 (N)—9.998 (SC) A; *β* = 129.46—129.47°. Variations in *β* are insignificant and do not exceed 3σ. Variations in other parameters are significant: *a*—up to 34σ, *b*—up to 14σ, *c*—up to 20σ. Parameters *a*, *b*, *c* reach maximum values in the SC series and minimum values in the N series.

Orthorhombic unit cell parameters of glushinskites obtained under a lack of Mn vary as follows: *a* = 12.675 (N)—12.705 (SC) A; *b* = 5.384 (N)—5.400 (SC) A; *c* = 15.405 (N)—15.433 (SC) A. Variations in parameters are significant: *a*—up to 30σ, *b*—up to 16σ, *c*—up to 28σ. Parameters reach maximum values in the SC series and minimum values in the N series.

Unit cell parameters of orthorhombic and monoclinic unit cells are related as *a_orth_* = *a_mon_*, *b_orth_* = *b_mon_*, *c_orth_* = 2*c_mon_*cos(*β_mon_*−90°).

As the Mg content in the solution increases up to Mg/(Mg + Mn) = 50%, parameters *a*, *c*, *β* of Mg lindbergites linearly increase (Figure 7). According to the angular coefficients of the corresponding regression lines (Table 2), the slowest increase occurs in the S series and the fastest in the NC series. For the N and SC series, the speed of growth is similar. On the contrary, in the same Mg concentration range, parameter *b* linearly decreases. The slowest decrease occurs in the S series and the fastest in the N and NC series. In the α′ setting, parameter *c* does not change as the Mg content grows, as shown by the example of the NC series (Figure 7d).

In the area of Mg lindbergite and Mn glushinskite co-existence, the *a*, *c* and *β* parameters of lindbergites and glushinskites obtained from the same solution are the closest, but the glushinskite parameters are significantly larger. Parameters *b* of these solid solutions are nearly equal (Figure 7). In the S series, as Mg/(Mg + Mn) in solution increases from 50 to 70%, the increase rate of *a*, *c* and *β* increases sharply (Figure 7a). The decrease rate of parameter *b* stays the same. For Mn glushinskites of this series, parameters *a*, *c*, *β* tend to increase insignificantly, and parameter *b* decreases with the increase in Mg content in the solution (and reduction in Mn content, respectively). In the case of other series, the width of the co-existence area does not allow us to evaluate trends of variation in the lindbergite and glushinskite lattice parameters.

Parameters *a*, *c*, *β* of Mn glushinskites (*a*, *c*, *β*—monoclinic cell, *a*, *c*—orthorhombic cell, as shown by example of NC series, Figure 7d) outside of the transition area do not change significantly and are close to the parameters of Mn-free glushinskite (Figure 7). Besides the NC series, the rate of the parameter *b* decrease is significantly higher than that for Mg lindbergite. The decrease is the fastest in the S series and the slowest in the NC series (Table 2). In the case of the N series, parameter *b* of Mn glushinskites cannot be determined due to the absence of *hkl, k* ≠ 0 reflexes, which are responsible for this parameter (see Section 4.3.1).

### 2.2. Scanning Electron Microscopy and EDX Spectroscopy

#### 2.2.1. Chemical Composition of Precipitates and Its Dependence on Solution Composition

The element composition of crystal intergrowths varies in all series of syntheses as the Mg/(Mg + Mn) ratio in solution changes (Table S3). In the estimation of the chemical composition of lindbergites and glushinskites, data on impurity phases (orthorhombic Mn oxalate and falottaite) were excluded. Mg/(Mg + Mn) ratio in these phases did not exceed 5%.

**Figure 8:**
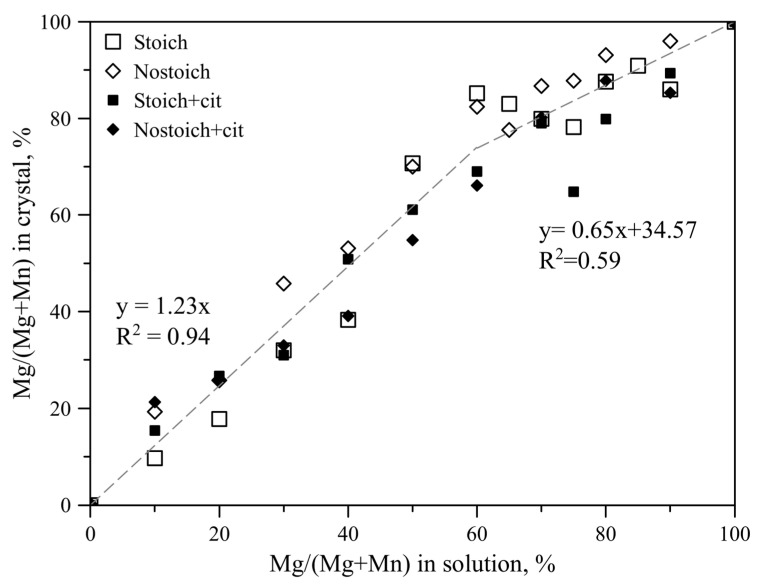
Dependence of Mg content in crystalline solid solutions (Mg, Mn)C_2_O_4_·2H_2_O on its content in solution. Average values are given.

On the “solution–solid” diagram (Figure 8), it is possible to single out two ranges that differ in the angular coefficients of regression lines and the significance of correlation coefficients depending on the experimental data’s dispersion. For Mg/(Mg + Mn) in solution up to 60% (lindbergite formation range), the linear correlation between Mg content in solution and solid is significantly stronger (R^2^ = 0.94) than for higher Mg concentrations (R^2^ = 0.59) where glushinskite starts to form. The angular coefficient of regression line for the first range is more than 1, and it is less than 1 for the second range.

Obviously, the maximum Mg concentration in the presence of citrate ions (66–69%, SC and NC series) is significantly lower than in their absence (82–85%, S and N series) at the inflection point.

The revealed regulations of correlations between the composition of solutions and crystal intergrowths (Table S3, Figure 8) allowed us to estimate the maximum content of Mg^2+^/Mn^2+^ in lindbergites/glushinskites (Table 3) in the case of single-phase precipitates (see Section 2.1.1). Such a method of estimation does not allow one to divide the effects of ionic substitutions in crystal structures of forming phases and the adsorption of impurity ions on the surfaces of crystals.

The average Mg/(Mg + Mn) ratio in lindbergites varies from 39 to 71% when the Mg/(Mg + Mn) ratio in solution ranges from 40 to 50%.

The average Mn/(Mg + Mn) ratio in glushinskites varies from 22 to 35% when the Mn/(Mg + Mn) ratio in solution ranges from 25 to 40%.

Estimation of the impurity cations in lindbergites/glushinskites within the transition area is impossible since they form complex intergrowths and cannot be distinguished by SEM with EDX, as will be shown below.

#### 2.2.2. The Influence of Element Composition on Crystal Morphology

The morphology of the synthesized crystals and crystalline aggregates of lindbergite–glushinskite series significantly depends on the Mg/(Mg + Mn) ratio and follows similar regularities in all four series of syntheses.

**Figure 9:**
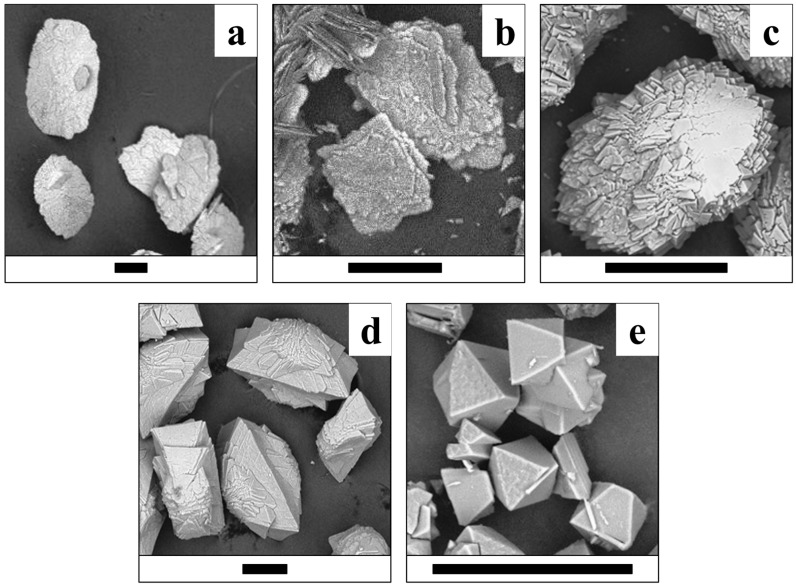
Crystals of (Mg, Mn) C_2_O_4_·2H_2_O solid solutions (S series) obtained at various Mg/(Mg + Mn) ratios in solution. (**a**)—0%; (**b**)–30%; (**c**)–70%; (**d**)—80%; (**e**)—100%. Black bar is 100 μm.

**Figure 10:**
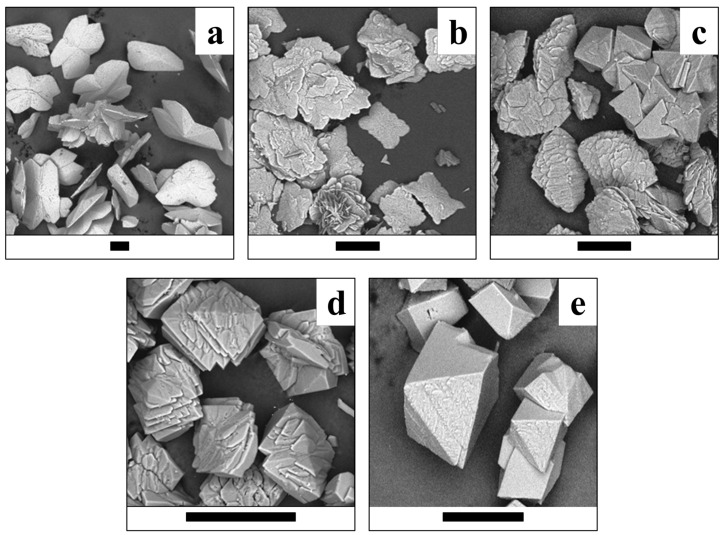
Crystals of (Mg, Mn) C_2_O_4_·2H_2_O solid solutions (N series) obtained at various Mg/(Mg + Mn) ratios in solution. (**a**)—0%; (**b**)—30%; (**c**)—65%; (**d**)—70%; (**e**)—100%. Black bar is 100 μm.

**Figure 11:**
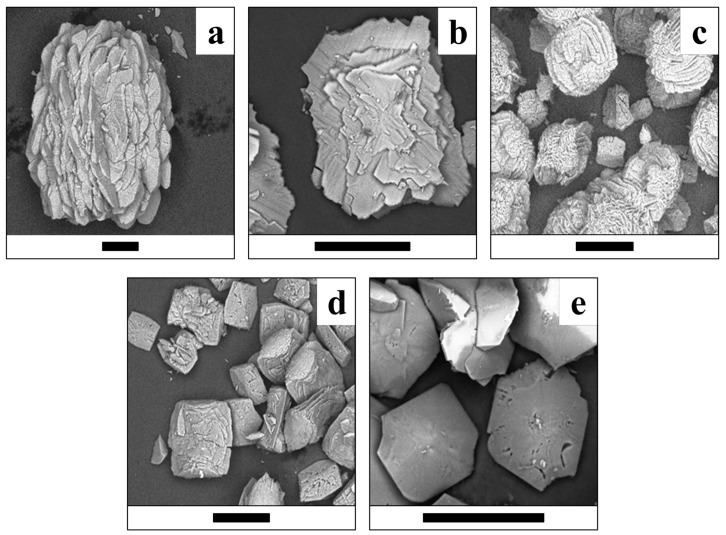
Crystals of (Mg, Mn) C2O4·2H2O solid solutions (SC series) obtained at various Mg/(Mg + Mn) ratios in solution. (**a**)—0%; (**b**)—30%; (**c**)—75%; (**d**)—80%; (**e**)—100%. Black bar is 100 μm.

**Figure 12:**
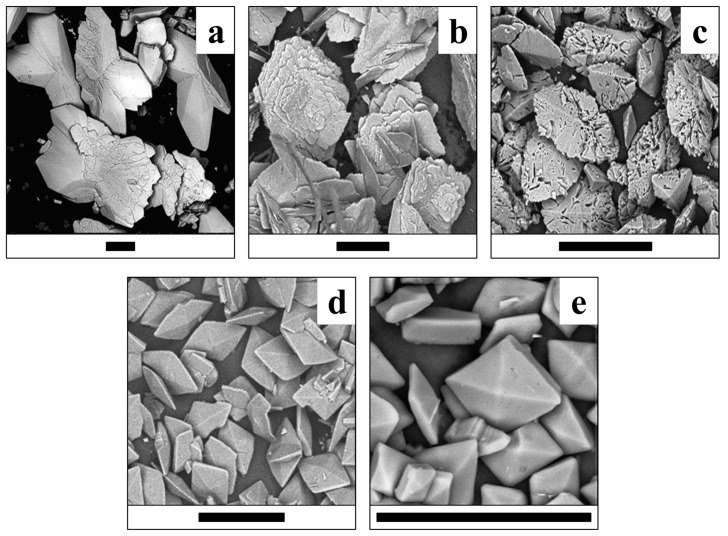
Crystals of (Mg, Mn) C_2_O_4_·2H_2_O solid solutions (NC series) obtained at various Mg/(Mg + Mn) ratios in solution. (**a**)—0%; (**b**)—30%; (**c**)—60%; (**d**)—80%; (**e**)—100%. Black bar is 100 μm.

While Mg is absent, lindbergite forms oval flat crystals with features of split growth (Figure 9a, Figure 10a, Figure 11a and Figure 12a). As the Mg content in solution increases (up to Mg/(Mg + Mn) = 50%), lindbergite crystallizes in rectangular, poorly faceted plates with numerous split flat subindividuals deviating from the middle of the main plate at a small angle. When the Mg/(Mg + Mn) ratio increases up to 60–75%, the plate crystals of lindbergite form clusters. The clusters become more and more complexed and pseudodipyramidal faces appear on them as the Mg content increases.

With a further increase in Mg content in solution, singular pseudodipyramidal glushinskite crystals are gradually formed by the faces of two orthorhombic prisms (8e, 9e, 10e, 11e). Moreover, a common pattern of crystal size change can be traced: from lindbergite crystals with a size of 500 μm and less to 20–70 μm crystals of glushinskite through various clusters of “transition areas” with 100–200 μm size. Such common alterations are characteristic for crystals of the ***S series*** primarily (Figure 9). This series also demonstrates the greatest deviations from linear growth regarding the Mg content in crystals when the Mg/(Mg + Mn) ratio in solution increases. For Mg/(Mg + Mn) in solution up to 40%, the growth proceeds with moderate speed (Mg/(Mg + Mn) in crystal = 38% for Mg/(Mg + Mn) in solution = 40%). Next, at Mg/(Mg + Mn) in solution = 40–60%, the Mg content in the crystal rises dramatically and amounts to 85% at Mg/(Mg + Mn) = 60% in solution, along with a rapid increase in Mg content dispersion in various crystal clusters (Mg/(Mg + Mn) = 56–84%). Then, in the S series, a decrease in the average Mg/(Mg + Mn) ratio from 85 to 78% takes place for Mg/(Mg + Mn) in solution = 60–75%. Finally, the increase in Mg content resumes and it reaches 90% at Mg/(Mg + Mn) = 90% in solution.

The morphology of crystals of the ***N series*** (Figure 10) differs from crystals of the S series. At first, crystals of lindbergite have a strongly flattened shape with smooth faces, and most of the crystals are represented by “swallowtail”-type twins (Figure 10a). Secondly, for Mg/(Mg + Mn) = 65% in solution, the formation of two types of crystal takes place: one of them is represented by flattened, elongated, pseudorhombic individuals (up to 150 μm in length) with splitting alongside flattening and almost absent side faceting (Figure 10c); the second one is represented by small, pseudopyramidal crystals (50–70 μm).

In the ***SC series***, the addition of citrate ions to solutions of the S series leads to new features in the crystal morphology (Figure 11). First of all, Mn-free glushinskite crystallizes as highly flattened, pseudodipyramidal plates (30–50 μm size) with smooth faces and clear signs of twinning (re-entrant angles in crystal faceting), as well as the emergence of narrow faces of the third rhombic prism (Figure 11e). Secondly, at Mg/(Mg + Mn) = 75% in solution, two types of crystal are formed: strongly split flat clusters (up to 150 μm) and small (up to 50 μm), nearly square plate crystals (Figure 11c).

In the ***NC series***, the addition of citrate ions in solutions of the N series leads to additional features in the crystal morphology as well. Mn-free glushinskite forms square, strongly flattened crystals (10–30 μm size) with faces of the third rhombic prism (Figure 12e). With the addition of Mn to the solution (Mg/(Mg + Mn) = 70–90% in solution), elongated, flattened, pseudodipyramidal crystals (50–100 μm length) without third prism faces (Figure 12d) are grown. In the transition area at Mg/(Mg + Mn) = 50–60% in solution, two types of crystal are formed: elongated, pseudorhombic crystals (up to 100 μm) having caverns on their faces and smaller (30–50 μm) and flattened pseudodipyramidal crystals (Figure 12c). Moreover, the reduction in the Mg concentration, at which the lindbergite–glushinskite transition occurs (from Mg/(Mg + Mn) = 70% to 60%), is characteristic for the NC series.

### 2.3. Thermodynamic Modelling

**Figure 13:**
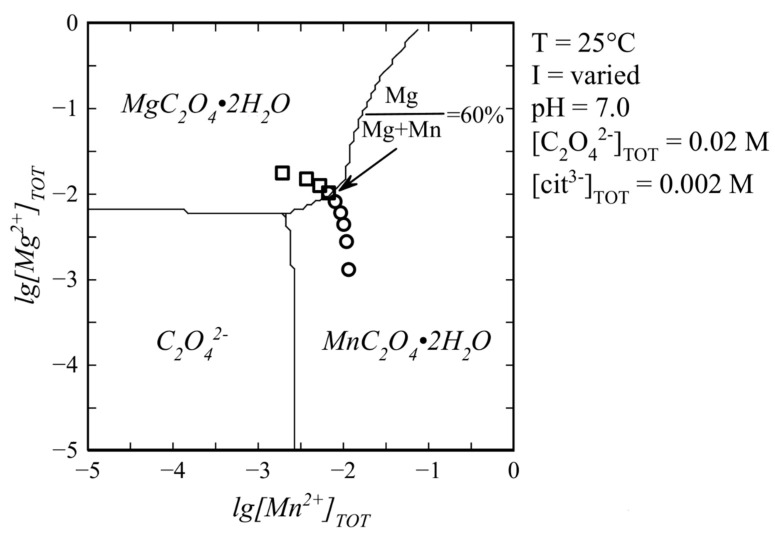
Typical predominance diagram for oxalate ion in water solution containing Mn^2+^, Mg^2+^ and citrate ion. Markers indicate Mg^2+^ and Mn^2+^ concentrations in syntheses of SC series: **□**—points within glushinskite field; ○—within lindbergite field.

According to the results of thermodynamic modelling (Figure 13), the dissolved oxalate ion is in equilibrium with Mg-free lindbergite at lg[Mn^2+^]_TOT_~−2.5 and with Mn-free glushinskite at lg[Mg^2+^]_TOT_~−2.2. All experimental points representing the initial concentrations in syntheses fall into the lindbergite or glushinskite field. Lindbergite and glushinskite are in equilibrium at Mg/(Mg + Mn) ~60%.

## 3. Discussion

Syntheses representing the conditions of crystallization of biofilm minerals have led to the formation of (Mn, Mg)C_2_O_4_·2H_2_O solid solution series. End-members of this series are analogues of biofilm minerals lindbergite and glushinskite.

The diffraction patterns of synthetic analogues of lindbergite MnC_2_O_4_·2H_2_O and glushinskite MgC_2_O_4_·2H_2_O differ significantly. Results of phase analysis (Section 2.1.1) have shown the formation of a monoclinic *α*-modification of Mn oxalate dihydrate (sp. gr. C2/c) in Mg-free syntheses. The monoclinic *α′*- and *α″*-MnC_2_O_4_·2H_2_O described in [7] are impossible to distinguish in the PXRD data. Taking into account the uncertainty of coordinate axis selection in the (010) plane in monoclinic crystals and equality *c_α′_*cos (*β_α_*_′_—90°) = *c_α″_*cos (*β_α″_*—90°) (see Section 2.1.2), the existence of *α′-* and *α″*-modifications is debatable and requires further single-crystal study.

In Mn-free syntheses, an orthorhombic *β*-modification of Mg oxalate dihydrate (sp. gr. *Fddd*) was formed. Obviously, the transition from monoclinic (*C*2/*c*) to orthorhombic (*Fddd*) unit cells is possible under the condition of equality:

(1) $$\frac{a_{mon}}{c_{mon}sin\left(\beta_{mon}-90{}^{\circ}\right)}=\frac{n}{m},\;$$

where *n* and *m* are natural numbers (see Figure 1c,d).

For Mg oxalate, such a transition is possible since *n/m =* 2.000 ± 0.002 (Table 4). For Mn oxalate, it is impossible since *n/m =* 2.070 ± 0.002 or 1.935 ± 0.005 (Table 4) and is an irrational number, which explains the unsuccessful attempt at the indexing of powder patterns in the *Fddd* space group.

As was pointed out previously, the crystal structures of *α*- and *β-*modifications (Figure 1) are very similar. The key differences are the sizes and deformations of *Me*^2+^O_4_(H_2_O)_2_ octahedra. Mn octahedra are significantly larger than Mg octahedra (<Mn-O> = 2.181 Å, <Mg-O> = 2.067 Å, Figure 1b). In the *β*-modification of Mg oxalate, octahedra are squeezed along the vector between the oxygens of H_2_O molecules (O2-O2), and four equatorial oxygens O1 form a rectangle. In the case of *α*-modification of Mn oxalates, octahedra are elongated along the vector between the oxygens of H_2_O molecules (O3-O3), and the equatorial oxygens 2O1 and 2O2 form a trapezium with a short O2-O2 side.

In orthorhombic *β*-MgC_2_O_4_·2H_2_O, chains of octahedra are packed in such a way that the vector between apical oxygens (O2-O2) is parallel to [100]. In Mn oxalate, larger Mn octahedra rotate around the [010] direction in such a way that the O3-O3 vector forms a ~1° angle with [100] direction in order to achieve denser packing (Figure 1c). This rotation eliminates the two-fold axis along the [100] direction, violates the ratio (1) between parameters *a*, *c* and *β* and does not allow a change to the orthogonal coordinate system (to higher orthorhombic symmetry).

Insignificant variations in the unit cell parameters of the synthesized and previously described analogues of lindbergites and glushinskites (Table 4) could be caused by minor variations in water content, which have not been studied yet. As hydrogen bonds are almost localized in the ac plane, alterations of water content can affect the *a*, *c* and *β* parameters. Moreover, differences in *c* and *β* in synthesized lindbergites and Mn oxalates in the PDF-2 database could be caused by uncertainty in the choice of *a* and *c* axes in monoclinic crystals (*α′-* and *α″*-modifications).

Synthesized analogues of biominerals are different not only in terms of crystal structure and symmetry but in terms of morphology as well: lindbergite forms plate intergrowths and glushinskite forms pseudo-octahedral crystals faced by two or three prisms. Excess oxalate ions cause “swallowtail” twinning in the case of lindbergite, and the presence of citric acid causes a crystal size increase, probably due to the formation of complexes between Mn and citric acid [22], leading to the lowering of the number of nuclei. Single, untwined crystals of Mg-free lindbergite can be obtained at Mn/C_2_O_4_ = 1 in the absence of citric acid.

In the case of glushinskite, an excess of oxalate ions causes an increase in crystal size. On the contrary, the presence of citric acid reduces crystals, flattens them and causes faces of the third prism to appear. The development of a third prism can be explained by the selective adsorption of citrate complexes, leading to the inhibition of the growth of this facet. A similar mechanism was previously revealed in Ca-Sr oxalates (analogues of weddellites) crystallizing from citrate-containing solutions [23]. Larger individual crystals of Mn-free glushinskite can be obtained with an excess of oxalate ions in the absence of citric acid.

Variations in the unit cell parameters of solid solutions along with Mg/(Mg + Mn) in solution (and, respectively, in crystals) allow us to reveal the regularities of Mg incorporation in lindbergite and Mn incorporation in glushinskite and analyze the regularities of lindbergite–glushinskite transition as the solid solution composition changes.

The incorporation of Mg in lindbergite is accompanied by a reduction in parameter *b*, which points out the squeezing of chains caused by the decrease in MeO_4_(H_2_O)_2_ octahedra (<Mn-O> = 2.181 Å, <Mg-O> = 2.067 Å [10,12]). Since the decrease is the fastest in lindbergites obtained in the presence of citric acid (Table 2), it is possible to assume that citric acid eases the incorporation of Mg in lindbergite. The increase in *a*, *c* and *β* can be explained by changes in the length and orientation of hydrogen bonds caused by the octahedra’s squeezing.

The broadening of lindbergite’s diffraction peaks and the appearance of glushinkite peaks after reaching Mg/(Mg + Mn) = 40–50% in solution (39–71% in crystal in area where glushinskite is not forming, Table 3) indicates that the lindbergite’s structure became unstable. The stoichiometric ratio of the main components *Me*^2+^/C_2_O_4_ = 1 (S and SC series) stabilize the lindbergite structure, and, accordingly, increase the limiting concentration of Mg in the crystal compared with series with the ratio *Me*^2+^/C_2_O_4_ < 1(N and NC series). In the presence of citric acid (*Me*^2+^/C_2_O_4_ ≤ 1, SC and NC series), magnesium partially forms complexes with citrate ions [24], preventing the adsorption of excessive Mg ions on the lindbergite crystals’ surface. EDX data include both Mg^2+^ ions on the Mn site and adsorbed on crystal faces. Thus, the limiting concentration of magnesium in lindbergites of the analyzed area, obtained in the presence of citric acid (Mg/(Mg + Mn) ~ 60%, Table 3), is closer to reality, since the effect of adsorption is less. The further incorporation of magnesium leads to the amorphization of lindbergite and its decay, which is accompanied by crystal splitting (Figure 9c, Figure 10c, Figure 11c and Figure 12c). In the area of co-existence of lindbergite and glushinskite, the concentration of Mg in lindbergite is even higher (reaching maximum of 70–80% in S series).

On the contrary, the incorporation of manganese in glushinskite results in an increase in the *b* parameter, which is explained by the expansion of chains caused by the increase in octahedra (<Mn-O> = 2.181 Å, <Mg-O> = 2.067 Å [10,12]). The almost or complete absence of *hkl, k≠0* peaks in the PXRD patterns of Mn-containing glushinskites indicates violations of the long-range order along the [010] direction. This effect is the most prominent in the system with Me^2+^/C_2_O_4_ < 1 (N series, Figure 5), where *k ≠ 0* peaks are completely absent, reflecting the non-periodicity along [010], which could be caused by the irregular alternation of Mg/Mn octahedra and oxalate ions in chains. Thus, unlike the three-dimensional ordered structure of Mg lindbergites, the crystal structure of Mn glushinskites is characterized by violations of the long-range order of various degrees and becomes 2D periodic in the system with ratio *Me*^2+^/C_2_O_4_ < 1.

The broadening of glushinskite’s PXRD peaks and the appearance of lindbergite peaks upon reaching Mn/(Mg + Mn) = 25–40% in solution (22 –34% in crystal in area where lindbergite is not forming, Table 3) indicates that the glushinskite structure has become unstable at a much lower impurity concentration compared with lindbergite. At the main component ratio Me^2+^/C_2_O_4_ = 1 (S and SC series), the limiting values at Mn/(Mg + Mn) in solution (25 and 30% respectively) and in crystal (22 and 31%) are almost equal. The ratio of the main components Me^2+^/C_2_O_4_ <1 (N and NC series) renders the entrance of Mn in crystals more difficult: Mn/(Mg + Mn) = 35 and 40% in solution gives 22 and 34% in crystal, respectively. The presence of citric acid increases the limiting concentration of Mn in glushinskite by 10% roughly, which is well explained by the earlier assumption of the formation of Mg–citrate complexes in a solution. The further entrance of Mn leads to the amorphization of glushinskite and its decay, accompanied by crystal splitting (Figure 9c, Figure 10c, Figure 11c and Figure 12c). In the lindbergite and glushinskite co-existence area, the Mn content in glushinskites is even higher (50–60%).

Generally, the limiting concentrations of Mn in glushinskite are significantly lower than the concentrations of Mg in lindbergite (by 20% roughly). We assume that the reason is the strict geometrical requirements for the preservation of the orthorhombic symmetry of glushinskite, as described above, and the higher rigidity of the structure overall.

The decomposition of solid solutions occurs at similar concentrations of cations Mg/(Mg + Mn) = 50–70%, which are in good agreement with the conditions of equilibrium between end-members, revealed by thermodynamic calculations (Mg/(Mg + Mn) = 60%, Figure 13). The area of co-existence of Mg lindbergites and Mn glushinskites contracts as the *Me*^2+^ content in solution decreases, and this occurs even more significantly with the presence of citrate ions. The way in which the lattice parameters change in this area indicates the leap in *α* ↔*β* transition (Figure 7). Thus, ionic substitutions in lindbergite–glushinskite series result in a first-order isodimorphic transition [25]. In the area of solid solution decomposition, the crystals of co-existing phases form joint intergrowths, preserving the morphological peculiarities of end-members.

## 4. Methods and Materials

### 4.1. Synthesis

Solid solutions of lindbergite–glushinskite series were synthesized in a water solution (500 mL volume) by adding the main components, MgCl_2_·6H_2_O, MnCl_2·_4H_2_O and their mixtures, into a solution of sodium oxalate (Na_2_C_2_O_4_) at room temperature (23–25 °C) and a pH of 4.0–7.3 for 7–36 days. The crystalline precipitate was filtered, washed with distilled water and dried at room temperature. Initial cation concentration ratios in solutions Mg/(Mg + Mn) varied from 0 to 100% with a 10% (5% in some cases) step.

Four series of syntheses were performed: at stochiometric ratios (Mn + Mg)/C_2_O_4_=1 (**S series**); at non-stochiometric ratios (Mn + Mg)/C_2_O_4_ <1 (**N series**); at (Mn + Mg)/C_2_O_4_=1 with the addition of citrate ions (0.002 M) (**SC series**) and at (Mn + Mg)/C_2_O_4_ <1 with the addition of the same amount of citrate ions (**NC series**). In the presence of citrate ions (SC and NC series), initial pH values in the range of 5.5–6.5 were achieved by adding NaOH. The addition of citrate ions allowed us to bring the crystallization conditions closer to those typical for biofoulings containing microscopic fungi, which are well known to produce a number of organic acids, including oxalic and citric [16].

### 4.2. Thermodynamic Modelling

Thermodynamic modelling was performed by plotting predominance diagrams for oxalate ions in lg[Mn^2+^]_TOT_—lg[Mg^2+^]_TOT_ axes using the “Database&Spana” (formerly “Hydra&Medusa”) software package based on the SOLGASWATER [26] and HALTAFALL [27] algorithms. The used values of solubility products were taken from [28,29].

### 4.3. Instrumental Methods

#### 4.3.1. Powder X-ray Diffraction (PXRD)

PXRD analysis was used to determine the phase composition of synthesis products and the unit cell parameters of Mg lindbergites and Mn glushinskites.

The survey was carried out by means of the Bruker D2 Phaser X-ray Diffractometer: voltage 30 kV, current 15 mA, CuKα irradiation, 2Θ = 5–70° with 0.02° step. The sample was crushed in a mortar and mounted on a low background sample holder from an ethanol suspension.

As previously published data pointed out the existence of Mg oxalate dihydrate in α- and β-modifications (*C*2/*c* and *Fddd* space groups, respectively), we tried indexing end-members in both space groups using INDX software (Tables S1 and S2) [30]. Lindbergite indexing was tried in three variants: for monoclinic α′ and α″ (sp. gr. *C*2/*c*, [7]) and for hypothetical orthorhombic modifications (sp. gr. *Fddd*). Lattice parameters for end-members were obtained through the Pawley method using TOPAS 5 software and the unit cell parameters of *α*-MgC_2_O_4_·2H_2_O [8], *β*-MgC_2_O_4_·2H_2_O [10], *α*’- and *α″*-MnC_2_O_4_·2H_2_O [7] as starting values.

At phase identification, lindbergite and glushinskite were distinguished by −202 and 200 reflexes (for *C*2/*c* indexing) in the 17–20° 2θ_CuKα_ range. A single peak matched these reflexes in the case of Mg oxalate, and a double peak in the case of Mn oxalate (Figure 1). The −4 0 2 reflex (for *C*2/*c* indexing) was used as well as it was significantly shifted to a lower angle area (from 2θ_CuKα_ ~30° to 2θ_CuKα_ ~28°) upon lindbergite–glushinskite transition.

The calculation of solid solution parameters, due to a lack of reliable initial values of parameters required by full-profile refinement methods, was performed by a custom algorithm iterating over all possible unit cell parameter combinations in the intervals *a* = 11.7–12.8; *b* = 5.3–5.7; *c* = 9.6–10.1 Å (or 14.9–15.6 Å for orthorhombic symmetry); *β* = 125–130° (or fixed at 90° for orthorhombic symmetry), with step 0.001 Å/0.01°. Then, the calculation of peak positions for the set of selected reflexes was performed (for 12 on lindbergite X-ray patterns and 10 on glushinskite X-ray patterns) and a search for the best matches with the experimental X-ray pattern was performed. The degree of matching was associated with the matching of calculated and experimental peak 2Θ values. The algorithm has shown good agreement with results on the end-members of solid solution obtained by full-profile refinement.

For solid solutions with glushinskite-like powder patterns, unit cell parameters were calculated in both monoclinic and orthorhombic variants. For solid solutions with lindbergite-like powder patterns, unit cell parameters were calculated only in monoclinic variants (for α′- and α″-modifications), since indexing in orthorhombic symmetry was not successful.

The standard error of unit cell parameter determination in single-phase precipitates by the used algorithm did not exceed 0.002 Å for linear parameters and 0.01° for the *β* angle. For solid solutions obtained in N series with Mg/(Mg + Mn) = 70–90%, parameter *b* was not possible to determine due to the absence of *hkl, k ≠* 0 reflexes.

With the presence of both lindbergite and glushinskite peaks, the error of unit cell parameter determination reached 0.01 Å and 0.1°, respectively, which was caused by the width and poor resolution of peaks of co-existing phases. In a number of cases, the unit cell parameters of one of the phases was not possible to determine at all, as will be shown later.

#### 4.3.2. Scanning Electron Microscopy (SEM) and Energy-Dispersive X-ray (EDX) Spectroscopy

SEM was used to investigate the morphology of the synthesized crystals. The research was carried out by means of a scanning electron microscope, the Hitachi S3400N (voltage 20 kV, probe current 1 nA, working distance 10–15 mm), equipped with an energy-dispersive attachment, the Oxford Instruments X-Max 20 energy-dispersive attachment. The attachment was used for the determination of the Mg/(Mg + Mn) ratio in crystals attached to carbon tape. Bulk Mn (purity 99.9%, Geller microanalytical laboratory) and crystalline MgO (purity 99.99%, Geller microanalytical laboratory) were used as standards.

## 5. Conclusions

The conducted crystal chemical studies have shown that despite similar crystal structures, the analogues of biominerals (lindbergite and glushinskite) belong to different modifications: lindbergite to monoclinic α-modification (sp. gr. *C*2/*c*), and glushinskite to orthorhombic *β*-modification (sp. gr. *Fddd*). Lindbergite–glushinskite transition occurs abruptly, indicating first-order isodimorphism. The limiting concentration of Mn in glushinskite is roughly 20% lower than that of Mg in lindbergite, which is caused by the higher rigidity of the glushinskite structure. The isomorphic capacity of lindbergite and glushinskite, the width of the transition and the equilibrium Mg/Mn oxalate ratio can be controlled by changing the Mg/Mn ratio in solution and introducing impurities—firstly, citric acid.

Structural interpretation of the symmetry differences showed that an increase in octahedra size leads to a decrease in the symmetry of the structure as a whole. Taking into account the obtained results, an assumption can be made about the symmetry of other oxalates of the humboldtine group, which, as was previously suggested, should be isotypical. Namely, we can assume the presence of an orthorhombic modification in Zn-bearing and two modifications in Fe-bearing oxalates (the ratio of octahedra sizes: <Mg^2+^-O> < <Fe^2+^-O> < <Mn^2+^-O> [6,11]).

These assumptions require further verification, but it is clear from the obtained results that they should be taken into account in biotechnologies aimed at the bioremediation/bioleaching of metals from media containing mixtures of these cations (Mg, Mn, Fe, Zn).

## Figures and Tables

**Table 2:** Coefficients of regression equations y = m + kx with x = Mg/(Mg + Mn) % in solution, y—one of unit cell parameters.

Series	Lindbergite								Glushinskite	
	*a*, Å		*b*, Å		*c*, Å		*β*, °		*b*, Å	
	k * 10^3^	m	k * 10^3^	m	k * 10^3^	m	k * 10^2^	m	k * 10^3^	y (100%) = m + 100k
S	3.7	11.974	−1.1	5.640	1.3	9.722	1.6	126.48	−5.0	5.386
N	4.6	12.007	−2.2	5.642	1.6	9.739	2.0	126.53		5.387
SC	4.6	11.998	−1.7	5.642	1.7	9.744	1.8	126.52	−3.4	5.400
NC	5.9	11.999	−2.1	5.640	2.0	9.734	2.3	126.55	−2.2	5.390

**Table 3:** Limiting concentrations of Mg/Mn in lindbergites/glushinskites within single-phase precipitates.

Series	Lindbergites, Mg/(Mg + Mn)%		Glushinskites, Mn/(Mg + Mn)%	
	Solution	Crystal	Solution	Crystal
S	50	71	25	22
N	40	53	35	22
SC	50	61	30	31
NC	40	39	40	34

**Table 4:** Unit cell parameters of synthesized lindbergites (sp. gr. *C*2/*c*) and glushinskites (sp. gr. *C*2/*c* and *Fddd*), and their natural analogues.

Origin	Series/Reference		*a*, Å	*b*, Å	*c*, Å	*β*, °	n/m
Lindbergite MnC_2_O_4_·2H_2_O							
Synthetic	S (our data)	*α″*	11.974(1)	5.640(1)	9.722(1)	126.48(1)	2.072
		*α′*	11.975(1)	5.641(1)	9.973(1)	128.40(1)	1.933
Synthetic	N (our data)	*α″*	12.007(1)	5.642(1)	9.739(1)	126.53(1)	2.071
		*α′*	12.008(1)	5.643(1)	9.989(1)	128.51(1)	1.931
Synthetic	SC (our data)	*α″*	11.998(1)	5.642(1)	9.744(1)	126.52(1)	2.069
		*α′*	11.996(1)	5.642(1)	9.982(1)	128.36(1)	1.936
Synthetic	NC (our data)	*α″*	11.999(1)	5.640(1)	9.734(1)	126.55(1)	2.070
		*α′*	11.999(1)	5.642(1)	9.974(1)	128.34(1)	1.939
Synthetic	PDF-2 #00-057-0602 (STAR) *		11.995(5)	5.632(2)	9.967(7)	128.34(4)	1.940
Synthetic	PDF-2 #01-086-6854 (STAR) *		11.939(5)	5.624(1)	9.703(3)	126.52(6)	2.068
Mineral	[5]		11.995(5)	5.632(2)	9.967(7)	128.34(4)	1.940
Synthetic (single crystal)	[12]		11.765(2)	5.655(1)	9.637(1)	125.84(1)	2.085
Glushinskite MgC_2_O_4_·2H_2_O							
Synthetic	S (our data)	*C*2/*c*	12.695(1)	5.386(1)	9.985(1)	129.46(1)	2.001
		*Fddd*	12.695(1)	5.389(1)	15.415(1)	90	
Synthetic	N (our data)	*C*2/*c*	12.675(1)	5.387(1)	9.978(1)	129.47(1)	1.998
		*Fddd*	12.676(1)	5.384(1)	15.405(1)	90	
Synthetic	SC (our data)	*C*2/*c*	12.709(1)	5.400(1)	9.998(1)	129.46(1)	2.000
		*Fddd*	12.705(1)	5.397(1)	15.433(1)	90	
Synthetic	NC (our data)	*C*2/*c*	12.695(1)	5.390(1)	9.983(1)	129.47(1)	2.000
		*Fddd*	12.698(1)	5.390(1)	15.413(1)	90	
Synthetic	PDF-2 #00-028-0625 (INDEXED) *, ****		12.675	5.406	9.984	129.45	1.998
Mineral	[3] **, ****		12.688	5.400	9.959	129.44	2.005
Synthetic (single crystal)	[10] ***		12.691(3)	5.394(1)	15.399(3)	90	

* Quality of database entry is indicated in brackets. ** Calculated with UnitCell [14] based on authors’ indexes and d-spacings. *** Crystal setting was changed as *a*→*b*, *b*→*a*, *c*→*c*. **** For published data, errors of unit cell parameters are not given if they were not present originally.

## Data Availability

Not applicable.

## Supplementary Materials

The supporting information can be downloaded at: https://www.mdpi.com/article/10.3390/ijms232314734/s1.
