# Supplementary material for: Solid Solutions of Lindbergite–Glushinskite Series: Synthesis, Ionic Substitutions, Phase Transformation and Crystal Morphology
Source: Int J Mol Sci. 2022 Nov 25;23(23):14734. doi: 10.3390/ijms232314734 (PMC9738142; doi:10.3390/ijms232314734)
Supplement: Supplementary file 1 [file ijms-23-14734-s001.zip › ijms-2029909-supplementary.pdf]

Table S1. Indexing of $MnC_2O_4 \cdot 2H_2O$ (NC series) in α' and α'' settings (C2/c space group).

d exp., Å for $MnC_2O_4{\cdot}2H_2O$ (NC series, our data)	*α'* setting, sp. gr. *C*2/*c*, *a* = 11.999Å, *b* = 5.642Å, *c* = 9.974Å, *β* = 128.34°*			*α''* setting, sp. gr. *C*2/*c*, *a* = 11.999Å, *b* = 5.640Å, *c* = 9.734Å, β = 126.55°*			d exp., Å for lindbergite [5]
	h	k	l	h	k	l	
4.809	-2	0	2	2	0	0	4.813
4.697	2	0	0	-2	0	2	4.704
3.911	0	0	2	0	0	2	3.909
3.673	-1	1	2	1	1	1	3.663
3.631	1	1	1	-1	1	2	3.626
2.997	-4	0	2	-4	0	2	2.998
2.821	0	2	0	0	2	0	2.816
2.682	-1	1	3	1	1	2	2.678
2.654	1<br>0	1<br>2	2<br>1	-1<br>0	1<br>2	3<br>1	2.654
2.408	-4	0	4	4	0	0	
2.371	2	0	2	-2	0	4	2.370
2.352	4	0	0	-4	0	4	2.352
2.287	0	2	2	0	2	2	2.285
1.996	-4	2	3	-4	2	1	1.993
1.949	-6	0	4	-6	0	2	1.947
1.925	-6	0	2	-6	0	4	1.925
1.845	-1<br>1	3<br>3	1<br>0	1<br>-1	3<br>3	0<br>1	1.843
1.824	-5	1	5	-3<br>5	1<br>1	5<br>0	
1.499	-8	0	4	-8	0	4	
1.373	-1<br>-2<br>6	1<br>4<br>2	6<br>1<br>0	1<br>-2<br>-6	1<br>4<br>2	5<br>1<br>6	

* Unit cell parameters of $MnC_2O_4 \cdot 2H_2O$, NC series, our data

Table S2. Indexing of $MgC_2O_4 \cdot 2H_2O$ (NC series) in *C*2/*c* and *Fddd* space groups.

d exp., Å for $MgC_2O_4 \cdot 2H_2O$ (NC series, our data)	Sp. gr. *C*2/*c*, *a* = 12.695Å, *b* = 5.390Å, *c* = 9.983Å, *β* = 129.47°*			Sp. gr. *Fddd*, *a* = 12.698Å, *b* = 5.390Å, *c* = 15.413°**			d exp., Å for glushinskite [3]
	h	k	l	h	k	l	
4.894	2<br>2	0<br>0	0<br>-2	2	0	2	4.89
4.717	1<br>1	1<br>-1	0<br>-1	1	1	1	
3.850	0	0	2	0	0	4	3.849
3.251	3<br>3	-1<br>-1	-1<br>-2	3	1	1	
3.174	4	0	-2	4	0	0	3.179
2.544	0	2	1	0	2	2	2.541
2.481	2	-2	1	2	2	0	2.492
2.449	4<br>4	0<br>0	0<br>-4	4	0	4	
2.381	2<br>2	0<br>0	2<br>-4	2	0	6	2.379
2.086	2<br>2	2<br>-2	1<br>-3	2	2	4	2.086
2.041	6<br>6	0<br>0	-2<br>-4	6	0	2	2.039
1.859	0	2	3	0	2	6	1.861
1.767	1<br>1	3<br>-3	0<br>-1	1	3	1	
1.643	3<br>3	-3<br>-3	-1<br>-2	3	3	1	
1.604	4<br>4	2<br>-2	1<br>-5	4	2	6	
1.588	8	0	-4	8	0	0	
1.528	6<br>6	-2<br>-2	-1<br>-5	6	2	4	1.526
1.461	5<br>5	-3<br>-3	-2<br>-3	5	3	1	
1.348	0	4	0	0	4	0	

* Unit cell parameters of $MgC_2O_4 \cdot 2H_2O$, NC series, our data

** $a_{orth} = a_{mon}$, $b_{orth} = b_{mon}$, $c_{orth} = 2c_{mon} * \cos(\beta_{mon}-90°)$

Table S3. Chemical composition of crystals of lindbergite – glushinskite solid solution series at various Mg/(Mg+Mn) ratios in solution by EDX.

Mg% in solution	Mg/(Mg+Mn) % in crystals, average	Standard deviation of Mg/(Mg+Mn) % in crystals		Mg% in solution	Mg/(Mg+Mn) % in crystals	Standard deviation of Mg/(Mg+Mn)% in crystals
**S series**				**N series**		
0.0	0.0	0.0		0.0	0.0	0.0
10.0	9.7	6.1		10.0	19.3	6.5
20.0	17.8	5.0		20.0	25.8	7.2
30.0	32.0	2.3		30.0	45.8	5.1
40.0	38.3	9.4		40.0	53.1	9.6
50.0	70.7	12.2		50.0	70.0	0.6
60.0	85.2	7.9		60.0	82.4	4.0
65.0	83.0	8.4		65.0	77.6	19.8
70.0	79.9	12.4		70.0	86.7	5.3
75.0	78.2	5.6		75.0	87.8	1.3
80.0	87.6	6.5		80.0	93.1	3.0
85.0	90.9	1.7				
90.0	86.0	8.9		90.0	96.0	1.4
100.0	100.0	0.0		100.0	100.0	0.0
**SC series**				**NC series**		
0.0	0.0	0.0		0.0	0.0	0
10.0	15.4	1.3		10.0	21.3	3.9
20.0	26.7	5.5		20.0	25.8	6.0
30.0	31.0	8.7		30.0	33.0	4.4
40.0	50.8	7.6		40.0	39.1	6.9
50.0	61.1	4.4		50.0	54.8	2.8
60.0	69.0	5.0		60.0	66.1	17.5
70.0	79.0	3.3		70.0	80.2	5.1
75.0	64.8	8.0				
80.0	79.9	6.7		80.0	87.8	5.2
90.0	89.4	0.8		90.0	85.3	14.4
100.0	100.0	0.0		100.0	100.0	0.0
